# “Even if I were to consent, my family will never agree”: exploring autopsy services for posthumous occupational lung disease compensation among mineworkers in South Africa

**DOI:** 10.3402/gha.v6i0.19518

**Published:** 2013-01-24

**Authors:** Audrey V. Banyini, David Rees, Leah Gilbert

**Affiliations:** 1 Chamber of Mines, Johannesburg, South Africa; 2 School of Public Health, Faculty of Health Sciences, University of the Witwatersrand, Johannesburg, South Africa; 3 National Institute for Occupational Health, National Health Laboratory Service, Johannesburg, South Africa; 4 Department of Sociology, University of the Witwatersrand, Johannesburg, South Africa

**Keywords:** mineworker autopsy, posthumous compensation, South Africa, consent for autopsy

## Abstract

**Context:**

In the South African mining sector, cardiorespiratory-specific autopsies are conducted under the Occupational Diseases in Mines and Works Act (ODMWA) on deceased mineworkers to determine eligibility for compensation. However, low levels of autopsy utilisation undermine the value of the service.

**Objective:**

To explore enablers and barriers to consent that impact on ODMWA autopsy utilisation for posthumous monetary compensation.

**Methods:**

In-depth interviews were conducted with mineworkers, widows and relatives of deceased mineworkers as well as traditional healers and mine occupational health practitioners.

**Results:**

A range of socio-cultural barriers to consent for an autopsy was identified. These barriers were largely related to gendered power relations, traditional and religious beliefs, and communication and trust. Understanding these barriers presents opportunities to intervene so as to increase autopsy utilisation.

**Conclusions:**

Effective interventions could include engagement with healthy mine-workers and their families and re-evaluating the permanent removal of organs. The study adds to our understanding of utilisation of the autopsy services.

South Africa has a statutory system for the post-mortem examination (autopsy) of cardiorespiratory organs of deceased mineworkers to determine their eligibility for compensation. The procedures (family consent, removal and transporting of organs) and benefits (which are largely financial) are governed by the Occupational Diseases in Mines and Works Act (ODMWA) (1). The actual pathological examinations and certification (the determination of the nature and severity of occupational disease) are centralised in Johannesburg, a city in Gauteng Province, which is distant from most mines and areas in which former mineworkers and their families live. Consequently, the organs are permanently removed and, after a period of storage, destroyed in Johannesburg. The organs are stored for a maximum of 2 years for re-examination in cases of appeal on the certification categories by the families. ODMWA certifications are categorised into either ‘no compensable disease’, ‘first degree’ or ‘second degree’, with the latter being the more severe occupational disease which carries a larger compensation payment.

The provisions of this Act are important as a large number of former mineworkers are not compensated in life (2–5). During 2001–2010, 311 deceased mineworkers who were not compensated while alive were found to have first-degree occupational lung disease and 2,426 second-degree disease. Additionally, 59 cases were upgraded from first to second degree. Applying the salary range used to calculate compensation payments ($3,340.50–$4,912.50), the 311 families were eligible for $1,038,895.50–$1,527,787.50 compensation in the first-degree category with an average of $4,126.50 per family. Similarly for those categorised as having second-degree disease, the 2,426 families were eligible for $24,260,000–$26,537,407.50 with an average of $10,469.38 per family. During the 10-year period, the 311 cases in the first-degree category and the 2,426 in the second degree were 2% and 18%, respectively, of all the autopsies performed.

Of particular poignancy in the South African context of impoverished rural communities is that despite the potential financial benefits for the families of deceased mineworkers, there is underutilisation of the autopsy service by black mineworkers. For example, during 2001–2010, The Employment Bureau of Africa (TEBA), which annually registers 71% of the total mine employees, recorded 28,265 in-service mineworkers’ deaths, all of whom were eligible for an autopsy (Herbet G, unpublished data, 2011). Yet, only 13,201 autopsies (i.e. 46.7% of TEBA-recorded deaths) were performed during this period (5). Assuming that the same 10-year average proportions of first- (2%) and second-degree (18%) certifications would have been applied to the 15,064 TEBA-recorded deaths, an additional 355 and 2,769 families would have been eligible for $1,185,869.62–$1,743,926 in the first-degree and $20,598,076.13–$30,291,695.50 in second-degree categories, respectively. It should be noted that these calculations of financial loss due to underutilisation of the autopsy underestimate the true losses because the number of deaths in non-TEBA-registered (in-service mineworkers) and retired mineworkers is unknown and are excluded from the calculations. Although money is only one aspect of defining poverty, the compensation award is up to 11 times the mineworkers’ monthly salary (6), and the award has been shown to offer a short-term financial relief to families of the deceased mineworkers (7).

For various reasons, the number of autopsies continues to fall despite the potential benefits of the procedure to families and medical science (8, 9). Some of the reasons for the decline include fear of mutilating the body, maintaining the body in the best condition possible, lack of knowledge about autopsy, belief that the examination during an autopsy inflicts further suffering to the body, the treating physician's lack of rapport with the deceased's family, lack of consensus among family members regarding the procedure, concern about funeral delays, dying at home as opposed to hospital or preference to preserve the dignity of the deceased over knowing the cause of death (10–14).

In South Africa, the ODMWA autopsies are declining especially among retired mineworkers. For example, of the total 4,255 ODMWA autopsies of black mineworkers during 2007–2010 (noting that there were 11,512 TEBA-recorded deaths), 19.6% were performed on retired mine-workers and the rest on in-service mineworkers (Ndlovu N, unpublished data, 2010). Insufficient knowledge of ODMWA provisions by the mineworkers, their relatives and health personnel in rural hospitals has been postulated as one of the causes of underutilisation (15). There is a dearth of research on this underutilisation. The aim of this paper is to fill this gap by exploring the enablers and barriers affecting ODMWA autopsy of black mineworkers and their families.

## Methods

This descriptive study used an exploratory, qualitative approach to obtain in-depth information from respondents. Such an approach is usually associated with the social constructivist paradigm which emphasises the socially constructed nature of reality. It is about recording, analysing and attempting to uncover the deeper meaning and significance of human behaviour and experience, including contradictory beliefs, attitudes and emotions. This approach was used with the aim of discovering the meanings that respondents attach to their choices, how they interpret situations, and what their perspectives are on autopsy-related issues. Qualitative studies provide an understanding of complex psychosocial issues (16), and the enquirer seeks insights into the meaning(s) attached to events by seeking disparate views, rather than looking to determine the most commonly held view (17). The advantage of qualitative methods in exploratory research is the use of open-ended questions and probing which gives respondents the opportunity to respond in their own words, rather than forcing them to choose from fixed responses, as quantitative methods do. Open-ended questions as used in this study have the ability to evoke responses that are meaningful and culturally salient to the participant, unanticipated by the researcher, and rich and explanatory in nature.

### Respondents

The respondents were former miners, relatives and widows of the deceased miners, in-service miners and others (traditional healers and occupational health practitioners). These categories of respondents were selected because of the particular perspective they were likely to bring. Miners and their families have a direct influence on autopsy utilisation, and traditional healers were thought to be able to offer a shared cultural perspective on attitudes towards autopsy and may also be in a position to influence behaviour. Occupational health practitioners were considered to be capable of offering a shared experience on the mineworkers’ attitudes towards autopsy as they had contact with miners and families during illness and death. In the main, informants were selected on the basis of their availability and accessibility. Individual informants were identified through community workers, other mineworkers and representatives of organised labour, except for traditional healers who were considered popular among mineworkers and each of whom represented a different cultural tradition.

The majority of retired mineworkers, relatives and widows were from Nongoma, a district of KwaZulu-Natal Province, which has a high density of mineworkers (Herbet G, unpublished data, 2011). Additionally, retired mineworkers living in five urban townships around Free State Province gold mines and in-service mineworkers from a Randfontein gold mine west of Johannesburg were selected on the basis of convenience, being relatively close to the base of the researchers.

### Data collection

The in-depth interview approach was selected for the study to explore individual or group meaning and experiences since human phenomena such as psychosocial responses are embedded in cultural patterns, and the inter-relationships among its components are complex, necessitating a flexible enquiry that allows concepts to emerge from the data being collected (16, 17). A semi-structured questionnaire was used to guide the interviews. Using open-ended questions, the researcher probed initial responses to gain in-depth insights. The questionnaire was piloted with three retired mineworkers and four in-service mineworkers and thereafter refined for content clarity. All of the interviews were conducted and audio taped by the first author, a medical doctor who speaks the vernacular languages of the respondents, in the respondents’ preferred language. Additional handwritten notes were also taken. Informants were free to stop and discontinue the interviews.

The issues covered in each interview were: labour history; mine medical surveillance and mine exit medical information; compensation legislation knowledge, ODMWA processes and benefits; and autopsy knowledge, perception and attitudes.

### Conceptual framework

**Fig. 1:**
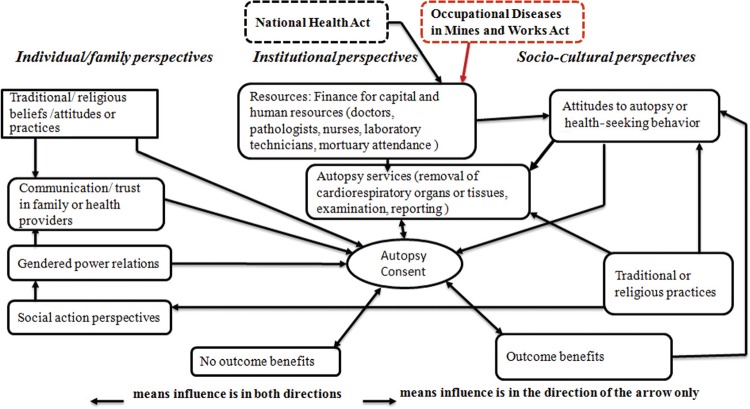
Conceptual framework of factors influencing clinical autopsy utilisation.

In the absence of adequate literature on barriers to an autopsy for the compensation or what encourages its utilisation, the literature on general clinical autopsy was used to formulate a conceptual framework for the study (Fig. 1). The identified potential factors playing a role in clinical autopsy utilisation were found to be at individual, institutional and community/societal levels. Factors at individual and socio-cultural levels were used to develop the semi-structured questionnaire to guide the interviews and the thematic analysis of the transcripts.

### Data analysis

All interview data were transcribed into English within 3 days of the interviews by the first author who speaks the vernacular languages and English to ensure recollection of discussions. Handwritten notes taken during the interviews were also included in the analysis. Key ideas generated were noted at the end of each interview. The notes for each informant were uploaded in MAXqda PC (2003), coded and grouped into thematic categories. Emerging themes were identified using the conceptual framework and their meaning interpreted.

### Number of respondents

The maximum number of respondents was set at 70 or whenever saturation of information was reached.

### Ethics

Informed consent was obtained from all respondents, and confidentiality was maintained. Relatives and widows who experienced the death of a mineworker within two years prior to the study were excluded to reduce psychological and emotional reactions invoked by discussing the death of their loved one. The study was approved by the Faculty of Humanities’ Human Research Ethics Committee (non-medical) in 2007, Ethical clearance number H070618.

## Results

**Fig. 2:**
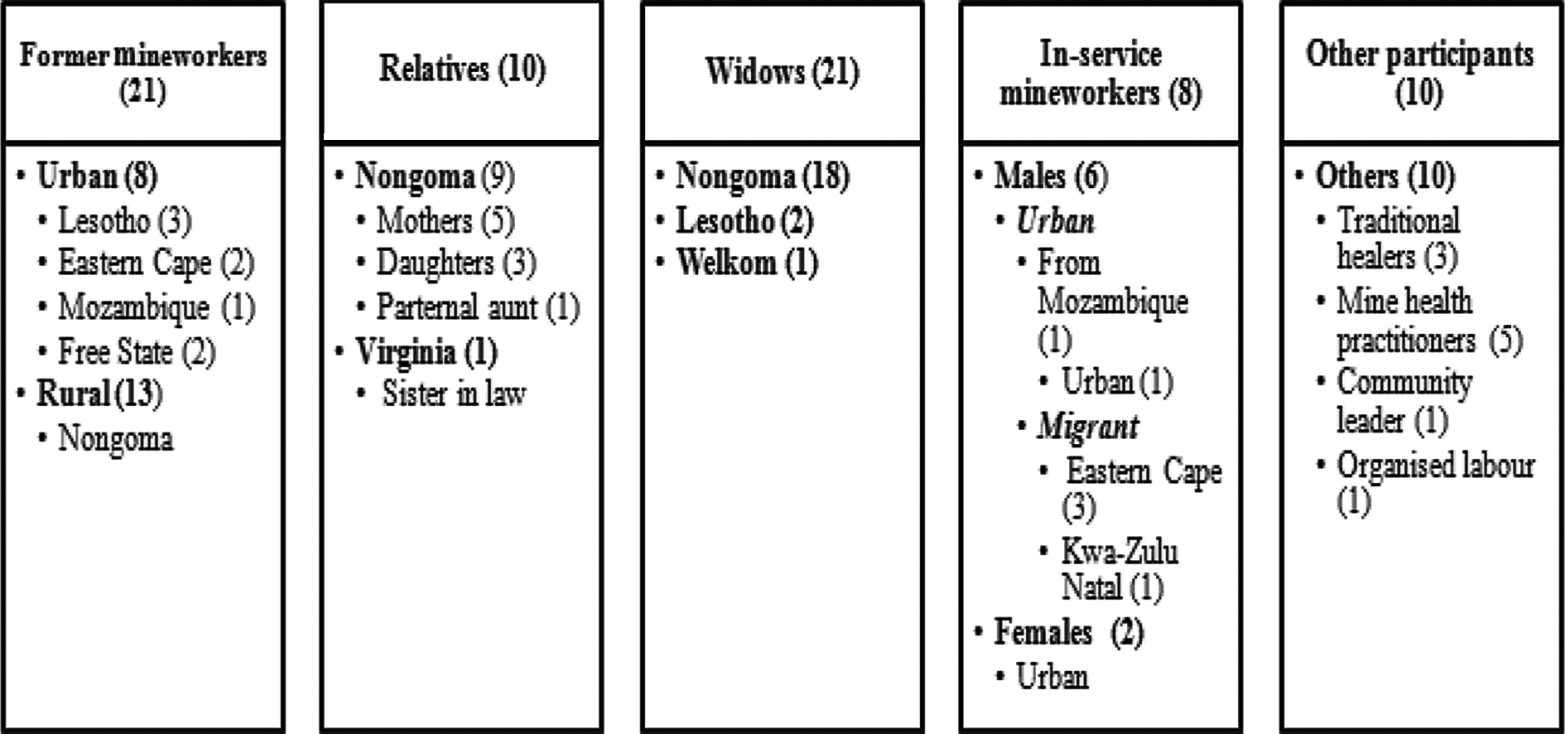
Key informants by category and number.

Seventy respondents were interviewed as shown (Fig. 2). Almost all of the mineworkers, their relatives and widows were unaware of the benefits and processes of ODMWA autopsy.

The results on the enablers and barriers impacting on autopsy utilisation were largely related to gendered power relations, traditional and religious beliefs, and communication and trust. For this reason, the findings are presented under these headings.

### Gendered power relations

According to respondents, during the bereavement period, widows observed a period of mourning and were excluded from funeral and burial rituals and decision-making. During this period of mourning, a widow's communication and contact with other family members would be limited until a pre-determined period and the person will be ritually cleansed. Some female respondents argued that even if they agreed to an autopsy, they would miss an opportunity to consent because of the cultural restrictions and taboos surrounding mourning. They articulated that a man's body belonged to his birth family and not to his wife or adult children. This implied that a man's patrilineal relations were stronger than to his wife. While the wives did not have decision-making power to give consent, patrilineal powers extended to paternal aunts who can communicate with ancestors. On the contrary, matrilineal relations in the absence of paternal relatives were conducive to autopsy. In addition, the absence of the deceased mineworker's family created an opportunity for the widow to give consent.

A widow from Nongoma said:

Autopsy, I will agree to find the cause of death … however, in my situation, he belongs to his family who has a final say …. I had no power around the death. You are only shown his body after the elder family women dressed him and they show you only his face. You are told not to look at face for long … you are mourning. You will know what is happening or get updates only if you have someone who really loves you …. You still don't have any power to question anything. My husband died in hospital and I took him there (silence) …. He died the following day and I was sent home to mourn him … yes as a wife you mourn. We used to hear about mineworkers who had autopsy at the mines …. this will be discussed, but their wives did not know about it … they did not see the scars.

A paternal aunt of the deceased mineworker in Nongoma articulated:

My brother's son died at the age of 31 years leaving a small child. He came back ill …. If I had knowledge about the autopsy, I will consent …. If I know where the parts from autopsy went, I would have agreed …. I would inform him and the ancestors through the sacrificial killing. I will consent because of the purpose and there is possibility that his kids, who are currently suffering financially, get a chance of compensation …. It is now the issue of the person not discussing the laws with the family and the doctors not talking. It is the current myth of young ones to believe that they will live longer than ugogo (grandparents) …. They do not communicate.

The respondents’ view on the widow's lack of decision-making power relating to her deceased husband was corroborated by traditional healers. According to them, the role of a wife during mourning is considered by the family to be a minor one. They explained that wives were alien to ancestral communication.

A traditional healer (from Gauteng) articulated:

If I die my family tradition and what is required is known by my family … they can inform my wife. They (my family) know which songs must be sung, the messages to be communicated to my ancestors and the rituals that must be performed in line with the family tradition and culture. The wife will not be able to sing this song for me, as she will not know the song. My wife would join after my family has completed the task ….

The gendered supremacy of the collective over the widow was reversed in the absence of the mineworkers’ family of origin and for those who live in a modern nuclear family structure.

A widow from the Welkom, Free State noted:

He was 53 years. He died of cancer of the lungs and breathed through a tracheotomy before he died. No autopsy was requested … he worked underground for many years. His parents passed on before him. I will consent to autopsy … you see I was alone and his family died earlier. I made all the decision surrounding his funeral and mourned him observing the family tradition.

### Traditional and religious beliefs

There were respondents who accepted autopsy because they believed that the soul dissociates from the body at death, and there were those who disagreed with the dissociation of the soul from the body. According to those who disagreed, cardiorespiratory organs were an engine that were crucial for the individual's existence in afterlife, resurrection, reincarnation and for becoming a good ancestor. They also believed that being buried without cardiorespiratory organs was similar to burying ‘an empty box’, and this will anger the ancestors and cause the deceased's soul to be rejected by them. The respondents described their fear of wrath and misfortunes from the ancestors and the deceased which included fear of their own deaths. Some respondents detailed their fear about their inability to meet the ancestral demands to return the cardiorespiratory organs for burial.

A former mineworker from Virginia, in the Free State, who was worried about the ‘empty box’, explained:

I don't agree with the autopsy … a person is these organs. They can be checked where he died … why send them away … my cardiorespiratory can't be sent away … because as people we have our cultural laws and beliefs. When we have our culture and you are buried apart from these organs …, the ancestors will not like it. Even if I can agree, the family won't agree that I am buried without these organs …. If the family consents when the person has died, he will come back to haunt them, cause them misfortune … while looking for his scattered parts. Traditionally we suffer from these decisions ….

The ‘empty box’ was corroborated by traditional healers.

Autopsy is not in our vocabulary as people. We believe that when a person dies, there should be no organs/parts removed. The person should be buried whole, so that when we reunite his body with his spirit …. We believe that when a person dies, he wakes up.

Some respondents argued that autopsy further inflicts pain on the deceased and disturbs their peace. This perception deterred the believers from accepting autopsy, as illustrated in the following citation.

An in-service mineworker from Mohlakeng and originally from Mozambique said:

My belief is that this (resurrection) will not be possible as this person was buried without complete parts …. I don't like this autopsy and will not agree to … when a person is dead, is asleep and autopsy is disturbing his/her peace … it is troubling the person and I will therefore not agree to this.

Some respondents argued that the soul and spirit were independent of the body. (The words soul and spirit were used interchangeably to denote a similar meaning.) According to respondents, once the soul and spirit departs from the body at death, the body and its parts are useless because the soul becomes ‘a complete being’ capable of conducting all functions. They expressed the view that the spirit is able to protect the living members of its family against misfortunes and advise them when consulted, and become a good ancestor and be reincarnated irrespective of the status of the body at death or burial. This is in contrast to the ‘empty box’ concerns of some respondents.

An in-service migrant mineworker from the Eastern Cape articulated:

I personally believe that ‘flesh’ is soil. Once spirit (umoya) leaves the flesh, the flesh has nowhere to go other than back to the soil. The soul of an ancestor does not go with flesh …. The ancestors are a soul/spirit. To me this same spirit is similar to that which is religiously preached …. It does not mean that when the flesh is incomplete, so is the spirit. The flesh is dead, there is nothing much that will be done by the flesh, it is dead and stone dead, it will never come back and what remains is to bury it.

A former mineworker from Nongoma expressed the following:

I can consent to autopsy because the body rots and is buried. My family may disagree with my acceptance of the autopsy …. I believe that the soul and spirit are complete, even if the person is maimed one way or the other. This has never changed in our tradition; it has always been known that once the person dies and the soul leaves the body, the soul can't draw back the flesh (body) and vice versa. This is a belief rooted in traditional values more than the born-again Christian concept which may vary …. I am not a born again. For an example, you die beheaded, you can't glue the pieces together, but the spirit is complete, not beheaded.

Some respondents believed the organ retention and autopsy were similar to commodification of the body parts. According to them, their role is caring for the body during life and up to the time of burial, as the soul can take care of itself. They were concerned that giving consent to ODWMA autopsy would be considered as sacrificing that responsibility.

A former mineworker from Nongoma's view was:

This autopsy is not correct, whether it is for compensation or not. It is like selling your body parts for money. I would not be motivated by money. Money finishes. It is like I sold my family, my child, never! …. So the doctor explains why autopsy should be done, to find the cause of death or there is disease and so what about it! Religion motivates one to take care of the soul more than the body … and rationalise that autopsy is ok …. Only modernised people agree to autopsy.

However, some respondents believed that autopsy would benefit them by elucidating the presence or absence of disease, confirm the cause of death and provide financial support for their families. Some believed that autopsy will provide closure on the mystery surrounding the cause of death and argued this mainly when the deceased were young.

An in-service mineworker from Mohlakeng, Randfontein said:

I am hearing about autopsy for the first time and I don't have any problems as it is ok to find out the cause of my death. I believe that it is right for the law to check whether I had disease … This may benefit my family. I was told about chest pick up for compensation when I was working at another mine … ever since I started here, I was never told anything. A deceased body has no further purpose in life and whatever can be done on my body to assist my family I can consent.

An in-service mineworker from Eastern Cape expressed:

I don't believe that being buried without lungs affects the position of an individual to be an ancestor (‘indlhozi’). The flesh rots; however, the spirit of that person remains alive and comes back to give the light to the family. The person can also appear during family dreams as a complete person and that body is no longer the flesh that was buried.

### Communication and trust

Respondents reported that the gendered power relations, family, traditional and religious fears to consent may be mitigated through proper communication with ancestors. Some respondents believed that communication will address the ‘empty box’ concern, deceased's anger and rejection by ancestors of an incomplete body and the suffering inflicted by the procedure. For the communication to be acceptable, the required rituals should be performed following autopsy acceptance by the mineworker and communication of this intention to the family while being healthy. According to respondents, communication to the ancestors is not acceptable if only carried out when the mineworker is ill.

An in-service mineworker from Eastern Cape said:

In my family culture, this autopsy may not be acceptable because they (ancestors) know that the person was complete when he died, and now he is buried being incomplete and tormented by this autopsy …. The ancestors will not recognise him …. This scenario changes if he has expressed his intention for autopsy in life because the family and he would have communicated this intention to ancestors in a proper way known to the ancestors. The ancestors will not disagree with him because they were consulted earlier about it …. However, if they consent without my prior knowledge and acceptance …. I will come and harass them (whoever gave consent).

A grandmother (from Nongoma) of deceased mineworker reiterated:

I don't like this autopsy (as you explain it). If I know, I can consent, and I will also remind the ancestors and him through the sacrificial killing. He will not cast a bad spell compared to him being buried only to be informed by the ancestors who do not welcome him due to missing parts that he was not aware of. If he told me about the autopsy and I did not inform the ancestors, he will trouble me.

Some respondents noted that for communication to be effective, trust among families and in the communities is required. They reported instances when men and their families mistrusted wives. According to them, there is a traditional belief that some husbands die because their wives killed them.

A former mineworker from Nongoma articulated:

I can consent to autopsy …. With regard to discussing autopsy with my wife for possible compensation, do I have to discuss this with her? The family can know about the autopsy but not my wife … (silence) … it is a problem. This law can also be told to the magistrate who can then implement it. I know of a friend of mine whose son died of stomach ulcers, do stomach ulcers kill? I believe the wife poisoned him so that she can get the money. The family found that he has been poisoned by his wife. How can an ulcer kill? This law must use the office of the magistrate. Women will finish the men and consent to autopsy if they know that there is going to be possible compensation …. In my culture, women do not have power to decide on this, only men have power for decisions. Women carry them out.

A former mineworker from Welkom said:

I believe that women are witches and that should they know that you have consented to ODMWA autopsy which has potential compensation, they can do anything sinister. You see, even when I was working … and now that I have retired due to ill health, I have never discussed anything with women, including money issues …. I am the breadwinner and her role is to receive any money I give her.

Some respondents expressed that autopsy should be treated the same way as items listed in an individual's will to ensure that the spouse and other members of their family are able to carry out the request if other family members questioned the procedure.

An in-service mineworker from Mohlakeng, Randfontein, explained:

I will consent to autopsy. I told my family that when I die, my wife must inform the mine that my cardiorespiratory organs can be removed for examination to check for occupational diseases and other organs if healthy be donated to help those who need them …. If my relative wrote in a will that he consents to autopsy I will carry it out because it is his wish and I would not want to disagree with his spirit and soul. His beliefs are not the same as mine. I would not want to disagree with his soul as an ancestor.

Some respondents said that there was a traditional belief that discussing death-related matters is challenging, and it may actually invite death to occur in their families. They noted that the fear to invite death limited individuals from discussing their post-death wishes. Respondents discussed their experiences with families that missed out on potential death benefits because of limited communication.

An in-service mineworker from Randfontein reiterated:

This autopsy is unusual. There are many people who will miss this compensation. It is in our culture that discussing matters relating to death is not a good thing as one could be calling for it (meaning inviting death) …. I don't believe that discussing it is challenging it. There are many people that I know who lost so much because their wives knew nothing and they had no information to argue their cases … they did not talk to their families …. There are many people I know who have lost everything they owned, which the family did not know or if they know, do not have the means of accessing these ‘materials’ or doing follow-up because the person told them nothing. This is what we pick up when we are in the villages during a funeral.

## Discussion

South African mineworkers have a high burden of occupational lung diseases (2–4), but many do not receive occupational compensation in life. Consequently the ODMWA autopsy provision is important, yet underutilisation of autopsy is substantial. The aim of this study was to explore the enablers and barriers affecting ODMWA autopsy utilisation by black mineworkers and their families to understand them so that effective interventions can be designed.

The study found that the enablers and barriers affecting ODMWA autopsy utilisation are diverse, complex and multifaceted and these were entrenched in cultural, religious or societal domains. The respondents rationalised their acceptance or rejection of autopsy within their own individual cultural or religious belief system, and these varied according to the individual's experiences, family beliefs and societal practices. The prerequisites to increase utilisation autopsy are significant and varied. Foremost, ODMWA autopsy process and the potential compensation benefits need to be empathetically communicated to all mineworkers, their families and communities while the mineworkers are still healthy to alleviate cultural barriers to consent. Trust concerning the ODMWA process must be established among the mineworkers, their families and society, and the purpose and the potential benefits must be understood by all parties. This finding suggests that central to building public and family trust, communication, awareness and knowledge should occur when mineworkers are healthy so that consent can be obtained easily at death. This perception presents an opportunity for policy makers and the mines to develop and implement intervention awareness and communication strategy, as this strategy would enable mineworkers and their families to perform rituals and would reduce the potential for a wife to be accused by the family and community of the murderer of her husband.

The requirement for consent is one reason for the decline in non-forensic autopsies (9). Although obtaining informed autopsy consent is to elicit the family's (and deceased patient's) religious, cultural and ethical sensitivities and determine what information is significant to decision making (19–22), the study found that family consent was deemed important to appease ancestors so that misfortunes are associated with causes other than the wrath of their ancestors. This finding is similar to other low-income settings that found that the initial decision making for informed consent was vested in the community rather than in the individual (20, 23).

This study established that retention of cardiorespiratory organs for re-examination in case of an appeal by families poses a major challenge. Burial without cardiorespiratory organs (‘empty box’) was not culturally acceptable to many respondents. Returning the organs to the body after examination was deemed culturally acceptable. Although this would address those that believed in the ‘empty box’, this intervention requires mobilisation of resources to ensure that examinations pose no delays to burial arrangement, as this may damage the trust by the family and community. Similar cultural beliefs were found in another study: burial with body parts missing or receiving an organ transplant (‘foreign’) was believed to prohibit an individual's transition to the realm of the ancestors by some black South Africans (23).

This study also found that the autopsy organ retention process in order to obtain the potential benefits was akin to commodification of body parts.

The various African religious and cultural beliefs found to be barriers to clinical autopsy; organ donation and this study are not unique. In Islam, autopsy and dissection of body parts for research are generally prohibited because of pre-destination belief and are akin to violation of human body sanctity (18, 22, 24). Unlike in Catholic and most Protestant religions, where autopsies are generally accepted, Hindu religion forbids it because of the belief in reincarnation (20). One study found that Judaic and Muslim families of the deceased gave consent to autopsy if it would benefit the family and communities’ lives or find the cause of death (24). In Muslim communities, post-mortem needle biopsy was accepted at a family and community level if it provided community benefits without violating family and cultural beliefs (24).

This study found that building awareness and increasing knowledge of families and communities while the mineworker was healthy to allow for ancestral ritual communication on the process rendered organ removal for examination acceptable to some, while others accepted only on-site examination and return of the organs for burial.

The findings from this study highlight that cultural factors present both barriers and opportunities for ODMWA autopsy. Unequal power relations control autopsy decisions and lead to exclusion of potential beneficiaries (widows and children). The fact that the cultural beliefs are not static or uniform suggests that increasing autopsy consent requires comprehensive communication and awareness intervention strategies aimed at an individual, family and community levels. The intervention strategies should not be once-off, but long term, and should address the cultural beliefs.

### Study limitations

Although respondents represented heterogeneous groups in three provinces affected by autopsy and saturation was reached in each group, the confined study settings may have limited the comprehensiveness of the issues identified and the generalisation of the findings to other groups. The respondents were selected by someone they knew and trusted. They may have said what they thought their contact and researcher would want to hear. While this bias could be real, the traditional healers and health practitioners corroborated the responses from the respondents, and this suggests that the responses were genuine.

### Policy implications

First, the findings that cultural beliefs influence ODWMA autopsy consent – and within the culture itself there are various interpretations – provide an opportunity to ensure that mineworkers, their families and the communities they come from or live in understand the purpose of autopsy in a manner that addresses these beliefs. The high burden of undiagnosed occupational lung diseases in life warrants this intervention and should be considered as a method to secure informed consent.

Second, decentralisation of autopsy examination so that organs can be examined and replaced before burial, thus removing the ‘empty box’ barrier, warrants consideration. However, replacing the organs for burial would remove the opportunity for re-examination during an appeal by families, and this issue needs consideration if decentralisation of autopsy is to be advocated. Additionally, the ODMWA autopsies’ data are comprehensive and have been the resource for both mineworkers’ occupational lung disease surveillance and research over many decades (25–28). Steps to preserve the ODMWA autopsy database would have to be put in place if decentralisation was to happen. Also, centralisation allows for a uniform and standardised pathological assessment of disease and the extent of disease. Steps to preserve these advantages would have to be implemented in a decentralised system.

## Conclusion

The study found not only barriers to autopsy but also cultural beliefs that may enable these barriers to be overcome. These findings may be generalisable to similar socio-cultural environments outside South Africa. Chief among the barriers was the requirement to obtain formal consent for autopsy within a biomedical framework, which clashed with certain socio-cultural beliefs. However, respondents reported that consent could be facilitated, for example, by mineworkers making their agreement to autopsy clear while being alive and by communicating this intention to ancestors – thus providing consent consistent with their belief system. ODMWA autopsy awareness and education should be geared towards families and communities of mineworkers. The enablers of autopsy could be used in campaigns to improve autopsy utilisation. More education for the mineworkers, their families and mining communities is required, while the mineworker is still alive to increase awareness and discussion of ODMWA autopsy's purpose and benefits.

Amending the ODMWA should be considered so that the current requirement of formal consent after death by the family is moderated, but ethical and legal aspects need to be taken into account. The current centralised autopsy procedure defined by ODMWA is a barrier to autopsy. The advantages and disadvantages of decentralising ODMWA autopsy to obviate a permanent removal of cardiorespiratory organs need investigation.

Further research is required to investigate the attitude of health care providers (nurses, traditional healers and medical practitioners) towards autopsy; to investigate the process of decentralising the autopsy examination and the impact it will have on appeals, standardisation and resources; and to examine the legal framework which would make ODMWA autopsy permissible without the current consent requirement, possibly under conditions similar to forensic medicine.
